# Brain metastases in patients with non-small cell lung cancer: the role of mutated-*EGFRs* with an exon 19 deletion or L858R point mutation in cancer cell dissemination

**DOI:** 10.18632/oncotarget.18509

**Published:** 2017-06-16

**Authors:** Shih-Hsin Hsiao, Yu-Ting Chou, Sey-En Lin, Ru-Chun Hsu, Chi-Li Chung, Yu-Rung Kao, H. Eugene Liu, Cheng-Wen Wu

**Affiliations:** ^1^ Program in Molecular Medicine, School of Life Sciences, National Yang-Ming University and Academia Sinica, Taipei 112, Taiwan; ^2^ Division of Pulmonary Medicine, Department of Internal Medicine, Taipei Medical University Hospital, Tapei 110, Taiwan; ^3^ Institute of Biotechnology, College of Life Science, National Tsing Hua University, Hsinchu 30013, Taiwan; ^4^ Department of Pathology, Wang Fang Hospital, Taipei Medical University, Tapei 11696, Taiwan; ^5^ Division of Thoracic Medicine, Department of Internal Medicine, School of Medicine and School of Respiratory Therapy, College of Medicine, Taipei Medical University, Taipei 110, Taiwan; ^6^ Institute of Biomedical Sciences, Academia Sinica, Taipei 11529, Taiwan; ^7^ Division of Hematology and Oncology, Department of Internal Medicine, Wan Fang Hospital, Taipei Medical University, Tapei 11696, Taiwan; ^8^ Graduate Institute of Clinical Medicine, Collage of Medicine, Taipei Medical University, Tapei 110, Taiwan; ^9^ Institute of Biochemistry and Molecular Biology, National Yang-Ming University, Taipei 112, Taiwan

**Keywords:** EGFR mutation, EGFR exon 19 deletion or L858R point mutation, non-small cell lung cancer, brain metastases

## Abstract

Non-small cell lung cancer (NSCLC) patients tend to develop brain metastases (BM), but the link between BM occurrence and driver mutations in NSCLC is not very clear. We explored whether activating mutations of epidermal growth factor receptors (*EGFRs*) in exon 19 deletion or L858R predict BM in NSCLC. A retrospective multivariable logistic regression analysis of 384 patients demonstrated that the presence of mutated-*EGFRs* was associated with overall BM (OR=2.24, P=0.001) compared to that of wild-type *EGFR* (WT-*EGFR*). Moreover, the time-to-event analysis model considering death as a competing risk revealed that, irrespective of survival, mutated-*EGFR*s predicted subsequent BM (SBM) in stage IIIB-IV patients (37.1% *vs*. 10.6%, HR=2.98, P=0.002) after adjusting for age (HR=2.00, P=0.012), gender, histological subtype, and smoking history. Notably, the younger mutated-*EGFR* subgroup was at a higher risk for SBM compared to the older WT-*EGFR* one (58.1% *vs*.10.9%, HR=6.57, P<0.001). Additionally, *EGFR* exon 19 deletion, despite having a slightly longer overall survival (20.6 *vs*. 14.2 months, P=0.368), was comparable to *L858R* mutation in predicting SBM (39.5% *vs*. 34.5%, HR=0.91, P=0.770). *In vitro*, the overexpression of mutated-*EGFR*s induced morphological changes towards a mesenchymal-like phenotype and promoted mobility in lung cancer cells. Clinically, mutated-*EGFR* NSCLC displayed a higher proportion of vimentin-positive expression (75.3% *vs*. 51.2%; P=0.007) and a shorter median time to SBM (23.5 months *vs*. not reached, P=0.017) than WT-*EGFR* NSCLC. These results suggest that NSCLC patients carrying mutated-*EGFRs* may require a higher frequency of brain imaging assessments than those with WT-*EGFR* to facilitate earlier SBM detection during follow-up.

## INTRODUCTION

Brain metastases (BM) occur in 20-40% of patients with non-small cell lung cancer (NSCLC) at some point during the disease course [1]. NSCLC is heterogeneous in terms of its histological subtypes and distinct driving oncogenes [2], which may affect the development of BM. Adenocarcinoma histology has been reported to be associated with BM from NSCLC. Epidermal growth factor receptor (*EGFR*) is one of the most common oncogenes, activating mutations of which drive tumor growth in NSCLC. The association between the *EGFR* mutation status and BM in patients with NSCLC has been noted in the past [3–6]. However, these findings have not been consistently observed. It is unclear whether the *EGFR* mutation status can predict the occurrence of subsequent BM (SBM) in advanced NSCLC or whether the high frequency of BM in *EGFR*-mutated NSCLC can be mainly attributed to the survival factor of *EGFR* mutations. Moreover, the rationales behind the above findings are not well determined. In addition, evidence suggests that *EGFR* exon 19 deletion-positive NSCLC is distinct from *EGFR* exon 21 (L858R) point mutation-positive NSCLC with regard to the tumor response to treatment and patient survival [7–10]. Nonetheless, the question regarding whether these two common subtypes of *EGFR* mutations have different impacts on the occurrence of BM in NSCLC has not been well addressed.

*EGFR* tyrosine kinase inhibitors (TKI), such as gefitinib, erlotinib or afatinib, preferentially target lung tumors with mutated-*EGFR*s, but not the wild-type *EGFR* (WT-*EGFR*) tumors, suggesting that the mutated- and WT-*EGFR*s have different oncogenic effects. *EGFR* protein expression was previously detected in various solid tumors, and *EGFR* expression correlated with cell migration/invasion in breast and oral cancer cell lines [11–13]. However, the ability of *EGFR* to enhance cell motility is ligand-dependent [11–13]. The participation of activating *EGFR* mutations in lung cancer cell mobility is unknown.

Therefore, in this study, we determined whether *EGFR* mutations, including the exon 19 deletion and L858R point mutation subtypes, predict the occurrence of the SBM in NSCLC patients, and characterized the role of activating *EGFR* mutations in lung cancer cell dissemination.

## RESULTS

### Flow chart of patient selection for analysis

**Figure 1:**
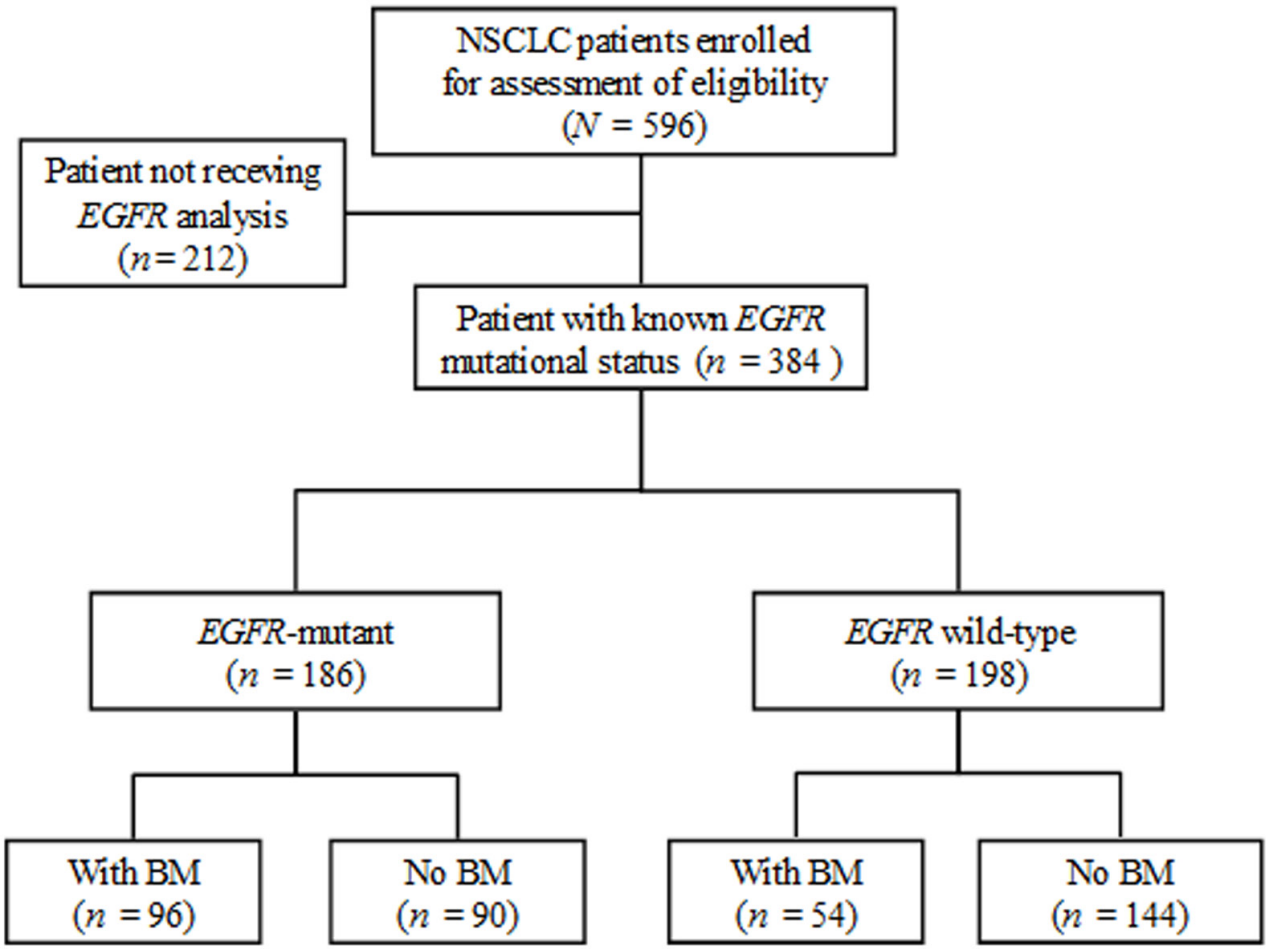
Flow chart of patient selection for further analysis Of the 596 non-small cell lung cancer (NSCLC) patients, 384 with a determined epidermal growth factor receptor (*EGFR)* mutation status, including 186 (48.4%, 186/384) with any or combined mutations of *EGFR* exon 18 to 21 and 198 with wild-type (WT) *EGFR*, were eligible for the identification of brain metastases (BM).

Of 596 NSCLC patients, 384 had a determined *EGFR* mutation status and were eligible for further analysis (Figure 1). This group had a median age of 68.1 years (interquartile range: 58.0-78.0 years) and a median follow-up time of 11.8 months (interquartile range: 3.9-24.8 months); 79 (20.6%) survived to the last follow-up.

Mutated-*EGFRs* were found in 186 (48.4%) of the 384 eligible patients (Figure 1), including an in-frame deletion in exon 19 (n = 79), a point mutation (L858R) in exon 21 (n = 97), and uncommon mutations (n = 10, 3 with an exon 18 point mutation, 6 with an exon 20 mutation, and 1 with an exon 18 and 20 mutation). The median OS of the mutated and WT patients was 20.6 months and 7.8 months, respectively (P < 0.001). The majority of the enrolled patients with stage IIIB-IV disease received cytotoxic chemotherapy and some received *EGFR*-TKIs (the first-generation) as the 1^st^-line care during the study period (Supplementary Materials and Methods). Of the 384 patients, 150 (39.1%) experienced BM, including 87 with BM at the diagnosis of their lung cancer and 63 with SBM during the follow-up period. Of 150 BM patients, 96 (64%) had mutated-*EGFR* and 54 had WT-*EGFR*.

### The presence of mutated-*EGFRs* is associated with overall BM

The patients’ characteristics at the time of their NSCLC diagnosis are shown in Table 1. Chi-square correlation analysis showed that young (55.0% *vs*. 32.6%), female (47.8% *vs*. 31.2%), never smokers (46.8% *vs*. 28.9%), patients with adenocarcinoma histology (42.2% *vs*. 23.4%), and patients with an advanced stage of lung cancer (43.0% *vs*. 18.0%) were more likely to have BM (P < 0.05). The overall cumulative incidence of BM was significantly higher in the patients with mutated-*EGFRs* than those with WT-*EGFR* (51.6% *vs*. 27.3%, respectively; P < 0.001). In details, 53.1% of the exon 19 deletion-positive patients and 49.5% of the L858R point mutation-positive patients experienced BM during their entire disease course.

**Table 1:** Baseline characteristics of 384 patients at the time of NSCLC diagnosis

Characteristics	Total (%)	Brain metastasis		*P*
		BM+ (*n* = 150)	BM- (*n* = 234)	
Gender				0.001
Male	202 (52.6)	63 (31.2)	139 (68.8)	
Female	182 (47.4)	87 (47.8)	95 (52.2)	
Age (years)				<0.001
≧60	273 (71.1)	89 (32.6)	184 (67.4)	
<60	111 (28.9)	61 (55.0)	50 (45.0)	
Smoking				<0.001
Current/former	166 (43.2)	48 (28.9)	118 (71.1)	
Never	218 (56.8)	102 (46.8)	116 (53.2)	
Histology				0.005
Adenocarcinoma	320 (83.3)	135 (42.2)	185 (57.8)	
Non-adenocarcinoma	64 (16.7)	15 (23.4)	49 (76.6)	
Stage				<0.001
IIIB - IV	323 (84.1)	139 (43.0)	184 (57.0)	
I - IIIA	61 (15.9)	11 (18.0)	50 (82.0)	
*EGFR*				<0.001
WT	198 (51.6)	54 (27.3)	144 (72.7)	
Mut	186 (48.4)	96 (51.6)	90 (48.4)	
*EGFR* Mut subtype (*n* = 186)				0.766
Exon 19	79 (42.5)	42 (53.1)	37 (46.9)	
L858R	97 (52.2)	48 (49.5)	49 (50.5)	
Uncommon	10 (5.4)	6 (60.0)	4 (40.0)	

NSCLC: non-small cell lung cancer; BM: brain metastases; *EGFR*: epidermal growth factor receptor; Mut: mutated; WT: wild type. The categorical data were presented as numbers (percentages) and the comparisons between groups used the chi-squared test. Non-adenocarcinoma group included squamous cell carcinoma, large cell carcinoma and NSCLC-not otherwise specified in this analysis. Uncommon mutations included 3 with an exon 18 mutation, 6 with an exon 20 insertion, and 1 with an exon 18 mutation and exon 20 insertion.

The multivariable logistic regression analysis, as shown in Table 2, revealed that the presence of mutated-*EGFR* was significantly associated with a higher overall cumulative incidence of BM, as compared to that of WT-*EGFR* (odds ratio (OR) = 2.24, 95% confidence interval (CI), 1.37-3.64, P = 0.001) after adjusting for gender (not significant), age (OR = 2.44, 95% CI, 1.52-4.00, P < 0.001), smoking history (not significant), and stage at lung cancer diagnosis (OR = 4.02, 95% CI, 1.94-8.32, P < 0.001). In terms of the specific subtype of mutated-*EGFRs*, both the presence of exon 19 deletion and the presence of L858R point mutation were significantly associated with BM compared to the presence of WT-*EGFR* (OR = 2.18, 95% CI, 1.19–4.00, P = 0.012, and OR = 2.13, 95% CI, 1.23–3.75, P = 0.009, respectively); however, the difference between the exon 19 deletion-positive and the L858R point mutation-positive groups was not statistically significant (OR = 1.03, 95% CI, 0.54-1.94, P = 0.939).

**Table 2:** Multivariable logistic regression analysis of the clinical factors for the overall occurrence of BM among 384 patients with NSCLC

Characteristics§	All patients (*N* = 384)				Stage IIIB - IV (*n* = 323)			
	Univariate		Multivariable		Univariate		Multivariable	
	OR (95% CI)	P	OR (95% CI)	P	OR (95% CI)	P	OR (95% CI)	P
Gender (male/female)	0.50 (0.33–0.75)	0.001	0.66 (0.37–1.17)	0.154	0.39 (0.25–0.61)	<0.001	0.53 (0.29–0.95)	0.033
Age (≧60/<60)	0.40 (0.25–0.62)	<0.001	0.41 (0.25–0.66)	<0.001*	0.43 (0.26–0.69)	0.001	0.44 (0.26–0.73)	0.002*
Smoking (ever/never)	0.46 (0.30–0.71)	<0.001	0.86 (0.47–1.58)	0.632	0.43 (0.27–0.68)	<0.001	0.94 (0.51–1.75)	0.848
Histology (adenocarcinoma/non-adenocarcinoma)	2.38 (1.28–4.43)	0.006	1.68 (0.83–3.40)	0.147	2.22 (1.17–4.22)	0.015	1.50 (0.73–3.09)	0.267
Stage (IIIB-IV/I-IIIA)	3.43 (1.72–6.84)	<0.001	4.02 (1.94–8.32)	<0.001*	–	–	–	–
*EGFR* (Mut/WT)	2.84 (1.86–4.35)	<0.001	2.24 (1.37–3.64)	0.001*	3.18 (2.01–5.04)	<0.001	2.34 (1.40–3.90)	0.001*
Pairwise comparison#								
Exon 19 / WT	3.03 (1.76–5.20)	<0.001	2.18 (1.19–4.00)	0.012*	3.63 (2.00–6.58)	<0.001	2.46 (1.29–4.68)	0.006*
L858R / WT	2.61 (1.58–4.33)	<0.001	2.13 (1.20–3.75)	0.009*	2.80 (1.62–4.82)	<0.001	2.19 (1.21–3.98)	0.010*
Exon 19 / L858R	1.16 (0.64–2.10)	0.627	1.03 (0.54–1.94)	0.939	1.30 (0.67–2.50)	0.434	1.12 (0.56–2.23)	0.741

BM: brain metastases; *EGFR*: epidermal growth factor receptor; Mut: mutated; WT: wild type; OR: odds ratio; CI: confidence interval. Non-adenocarcinoma group included squamous cell carcinoma, large cell carcinoma and NSCLC-not otherwise specified in this analysis. # Uncommon mutation was not shown in the *EGFR* pairwise comparison due the small number of patients. In addition, the pairwise comparison was made in another multivariable logistic regression model. §Category after the slash (/) was set as reference category; * indicated significant at *P* < 0.0125 level in the multivariable model.

### The presence of mutated-EGFR predicts a higher cumulative incidence of SBM

**Figure 2:**
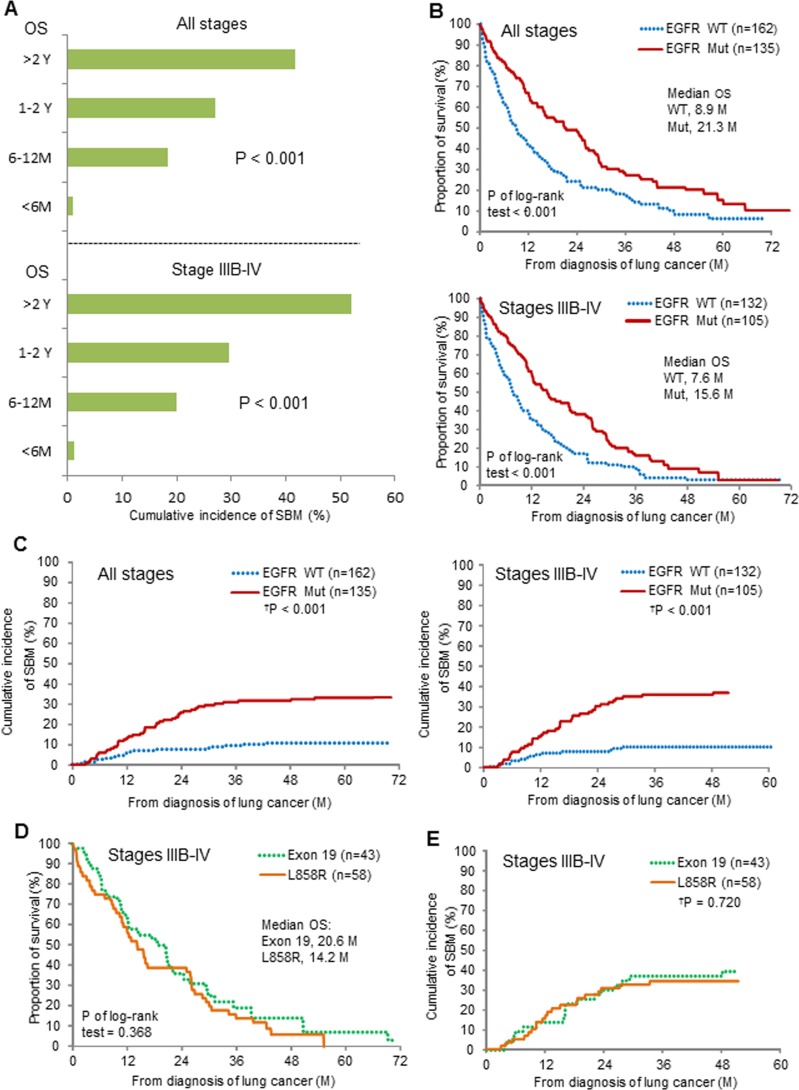
Correlation of mutated-EGFRs with subsequent brain metastases (SBM) in patients without BM at the diagnosis of NSCLC **(A)** The length of overall survival (OS) and the cumulative incidence of SBM. **(B)** *EGFR* mutation status and OS. **(C)** *EGFR* mutation status and SBM. **(D, E)** Exon 19 deletion and L858R point mutation, and OS and SBM. Comparisons regarding OS and SBM were done using log-rank test and time-to-event data analysis considering death as a competing risk (^†^P) (Fine and Gray's sub-distribution hazard model). Note, M = month; Y = year; Mut = mutated; WT = wild-type.

To test whether a favorable overall survival (OS) influenced SBM in NSCLC patients who did not have BM at the diagnosis of lung cancer (n = 297), we correlated the length of OS with SBM occurrence. We found that the length of OS was associated with the cumulative incidence of SBM (P < 0.001), which strikingly increased from 1% (1/96) in all-stage patients with OS less than 6 months to 41.7% (35/84) in those with OS longer than 2 years (Figure 2A, upper). A similar surge of SBM was observed in the patients stratified from IIIB-IV NSCLC with OS longer than 2 years as compared to those with OS less than 6 months (Figure 2A, lower). Kaplan–Meier analysis showed that *EGFR*-mutation status was associated with better survival outcomes in NSCLC (Figure 2B). To further determine whether the presence of mutated-*EGFR* predicted SBM, which is independent of the *EGFR* mutation-related better survival, we conducted a time-to-event analysis model considering death as a competing risk (Fine and Gray's sub-distribution hazard model) and found the cumulative incidence of SBM in the patients of all-stage NSCLC was 33.3% (45/135) in the mutated group and 11.1% (18/162) in the WT group, respectively (Hazard ratio (HR) = 3.0, 95% CI = 1.83-4.93, P < 0.001, Figure 2C left), and that in those of stage IIIB-IV NSCLC was 37.1% (39/105) in the mutated group and 10.6% (14/132) in the WT group, respectively (HR = 3.82, 95% CI = 2.07-7.06, P < 0.001, Figure 2C right). As to the stage I-IIIA patients primarily treated with surgery, the cumulative incidence of SBM between the mutated- and the WT-*EGFR* groups were not statistically different (25.0% (6/24) and 18.7% (3/16), respectively, P = 0.88, data not shown).

As to the comparison between the exon 19 deletion-positive and the L858R mutation-positive groups from stage IIIB-IV patients, there was a slightly longer median OS (20.6 *vs*.14.2 months, P of log-rank test = 0.368) in the former (Figure 2D), but no difference in the cumulative incidence of SBM (39.5% *vs*. 34.5%, HR= 1.12, 95% CI = 0.60-2.09, P = 0.720, Figure 2E) was observed between the two groups.

### The presence of mutated-*EGFR* is independently associated with SBM

To confirm whether the presence of mutated-*EGFR* was independently associated with SBM, multivariable analysis was performed. For stage IIIB-IV NSCLC patients, the presence of mutated-*EGFR* was significantly associated with the occurrence of SBM as compared to that of WT-*EGFR* (HR = 2.98, 95% CI, 1.50-5.93, P = 0.002) after adjusting age (HR = 2.00, 95% CI =1.16-3.45, P = 0.012) and other common demographic covariates (Table 3). Similar results were observed for both the exon 19 deletion-positive and the L858R point mutation-positive groups when compared to the WT-*EGFR* group (HR = 2.79, 95% CI, 0.36-1.25, adjusted P = 0.012 and HR = 3.08, 95% CI, 0.33-1.49, P = 0.002, respectively). Further analysis revealed that there was no difference for SBM occurrence between the exon 19 deletion and L858R point mutation groups (HR = 0.91, 95% CI, 0.47–1.74, P = 0.770).

**Table 3:** Multivariable analysis of the clinical factors for the occurrence of the subsequent BM among 297 patients without BM at the diagnosis of their NSCLC

Characteristics§	All patients (*N* = 297)				Stage IIIB - IV (*n* = 237)			
	Univariate		Multivariable		Univariate		Multivariable	
	HR (95% CI)	*P*	HR (95% CI)	*P*	HR (95% CI)	*P*	HR (95% CI)	*P*
Gender (male/female)	0.69 (0.44–1.07)	0.095			0.52 (0.30–0.88)	0.016	0.73 (0.38–1.40)	0.340
Age (≧60/<60)	0.49 (0.32–0.75)	0.001	0.51 (0.34–0.77)	0.001*	0.48 (0.28–0.83)	0.008	0.50 (0.29–0.86)	0.012*
Smoking (ever/never)	0.54 (0.33–0.87)	0.011	0.91 (0.56–1.46)	0.690	0.49 (0.28–0.89)	0.018	1.02 (0.50–2.10)	0.950
Histology (adenocarcinoma/non-adenocarcinoma)	3.20 (1.22–8.44)	0.018	1.96 (0.70–5.48)	0.200	3.08 (1.07–8.88)	0.037	1.74 (0.52–5.89)	0.370
Stage (IIIB-IV/I-IIIA)	1.34 (0.73–2.48)	0.350			–	–	–	–
*EGFR* (Mut/WT)	3.00 (1.83–4.93)	<0.001	2.45 (1.42–4.22)	0.001*	3.82 (2.07–7.06)	<0.001	2.98 (1.50–5.93)	0.002*
Pairwise comparison#								
Exon 19 / WT	3.05 (1.73–5.39)	<0.001	2.33 (1.25–4.35)	0.008*	4.00 (1.99–8.04)	<0.001	2.79 (0.36–1.25)	0.012*
L858R / WT	2.79 (1.60–4.87)	<0.001	2.41 (1.34–4.31)	0.003*	3.57 (1.80–7.09)	<0.001	3.08 (0.33–1.49)	0.002*
Exon 19 / L858R	1.09 (0.66–1.81)	0.720	0.97 (0.59–1.59)	0.900	1.12 (0.60–2.09)	0.720	0.91 (0.47–1.74)	0.770

BM: brain metastases; NSCLC: non-small cell lung cancer; *EGFR*: epidermal growth factor receptor; Mut: mutated; WT: wild-type; HR: hazard ratio; CI: confidence interval. Non-adenocarcinoma group included squamous cell carcinoma, large cell carcinoma and NSCLC-not otherwise specified in this analysis. # Uncommon mutation was not shown in the EGFR pairwise comparison due to the small number of patients. In addition, the pairwise comparisons were made in another multivariable model. § Category after the slash (/) was set as reference category; * indicated significant at *P* < 0.0125 level in the multivariable model. The association between the presence of mutated-*EGFRs* and subsequent BM was tested using a time-to-event analysis considering death as a competing risk (Fine and Gray's sub-distribution hazard model).

### The mutated-*EGFR*s promote cancer cell dissemination

**Figure 3:**
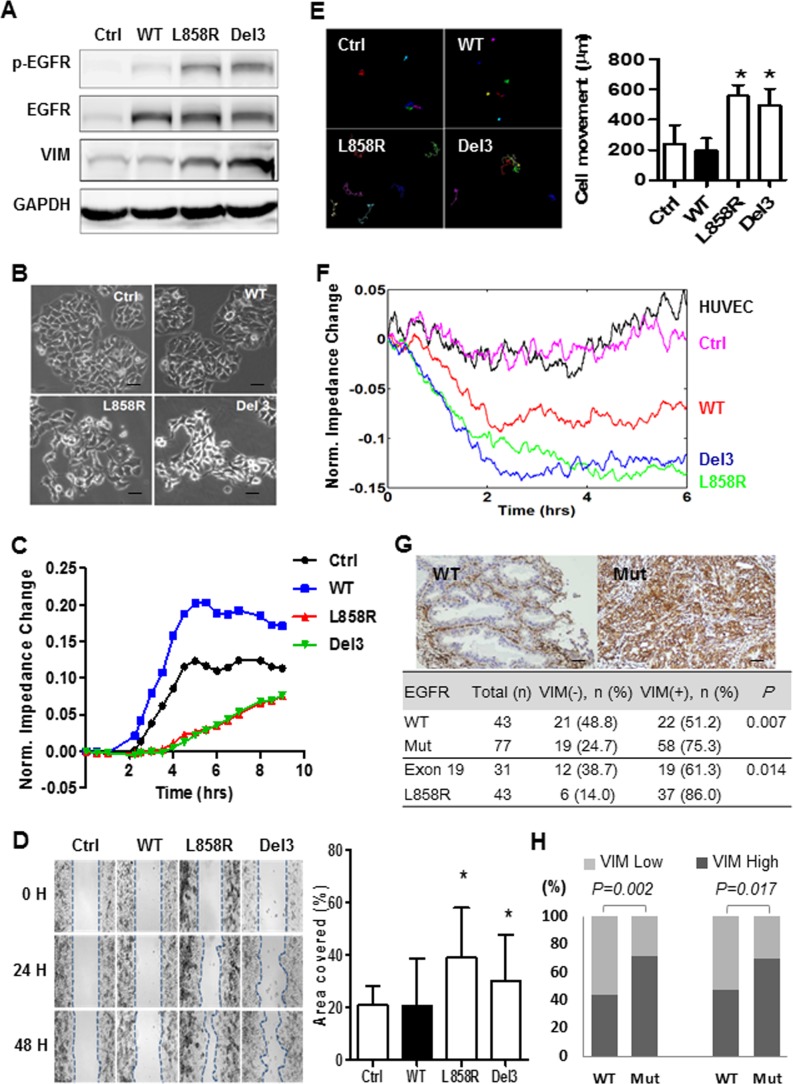
The mutated-EGFRs, exon 19 deletion and L858R point mutation, induce a mesenchymal-like phenotype and encourage cancer cell dissemination **(A)** Western blot analysis of phosphorylated *EGFR* (p-EGFR), total EGFR (EGFR), vimentin (*VIM*) and GAPDH in H1437 cells infected with lentiviral vectors encoding wild-type (WT) and mutated-*EGFR*s (L858R, a point mutation in exon 21; Del 3, an in-frame deletion in exon 19 of *EGFR* tyrosine kinase domain) or an empty control vector (Ctrl). **(B)** Representative phase-contrast images of H1437 cells infected with lentiviral vectors encoding WT and mutated-*EGFR*s (L858R or Del 3) or empty control vector (magnification = 100X, scale bar = 100 μm). **(C)** Electric cell-substrate impedance sensing (ECIS) analysis to monitor the changes in cell impedance as a result of the *EGFR* functional responses in H1437 cells infected with lentiviral vectors encoding WT and mutated-*EGFR*s or an empty control vector. **(D)** Representative images of the wound healing assay for H1437 cells infected with lentiviral vectors encoding wild-type (WT) and mutated-*EGFR*s (L858R or Del 3) or the empty control (Ctrl) vector (upper). The dotted lines indicate the wound edge at 0, 24 and 48 hrs, respectively (magnification = 50X). Quantitative analysis for the cells migrated during 48 hours (lower) (student *t*-test). * indicated significant at P < 0.05. **(E)** Representative images of cell tracking assay for H1437 cells infected with lentiviral vectors encoding WT and mutated-*EGFR*s or an empty control vector (upper). The colored lines represented the individual tracks of the motile cells (magnification = 100X, scale bar = 100 μm). Quantitative analysis for cell tracking assay (lower) (student *t*-test). * indicated significant at P < 0.05, ** indicated significant at P < 0.01. **(F)** An ECIS assay to monitor the changes in cell impedance in HUVEC cells as a result of the invasion of H1437 cells infected with lentiviral vectors encoding WT and mutated-*EGFR*s or an empty control (Ctrl) vector. **(G)** Association between vimentin (*VIM*) expression and *EGFR* mutation status in patients with lung adenocarcinoma. Representative pictures of the immunohistochemical analysis of *VIM* expression in adenocarcinoma specimens. A *VIM*-negative, WT *EGFR* adenocarcinoma case (upper left) and a *VIM*-positive, mutated-*EGFR* (Mut) adenocarcinoma case (upper right) are shown. The pictures were scanned at x 40 magnification (scale bar = 100 μm) and the size was adjusted to the screen. The distribution of *VIT* expression in the lung cancer specimens from the mutated- and WT-*EGFR* groups was compared using Fisher's exact test (bottom). **(H)** Correlation analysis of *VIM* expression with *EGFR* mutation status in mutated- and WT *EGFR* lung adenoacrinomas (Chi-square test). The sources of *VIM* expression levels of lung cancer and the corresponding clinicopathological parameters were downloaded from Chitale et.al. Memorial Sloan-Kettering Cancer Center (http://cbio.mskcc.org/public/lung_array_data/) (Left) and TCGA (https://tcga-data.nci.nih.gov/docs/publications/luad_2014/) (Right).

To study the potential effect of the mutated-*EGFR*s on lung cancer progression, mutated *EGFR* [L858R (a point mutation in exon 21) or Del 3 (an in-frame deletion in exon 19)] or the wild-type (WT) one was introduced into H1437 (non-mutated) lung adenocarcinoma cells via lentiviral infection (Figure 3A). The ectopic expression of mutated- but not WT-*EGFR*s induced a morphological change from an epithelial phenotype to a spindle-like morphology (Figure 3B and Supplementary Figure 1A). Additionally, the electric cell-substrate impedance sensing (ECIS) analysis revealed that the mutated-*EGFR*s attenuated the impedance, indicating that the mutated-*EGFR*s inhibit barrier properties in lung cancer cells (Figure 3C). A wound healing assay showed that the mutated-*EGFR*s promoted cell migration, compared to the WT-*EGFR* (Figure 3D and Supplementary Figure 1B). In support of this notion, the cell-tracking assay showed that migration of H1437 cells were promoted by mutated-*EGFR*s but not the wild-type one (Figure 3E). Moreover, an endothelial cell-based invasion analysis was adopted to examine the metastatic potential of mutated-*EGFR* cells. We observed that H1437 cells carrying the mutated-*EGFR*s invaded more profoundly into the endothelial cells, thus attenuating the impedance of endothelial cells as compared to their wild-type counterparts (Figure 3F). Immunoblotting showed that the mutated-*EGFR*s not only elevated the levels of phosphorylated *EGFR* but also increased vimentin expression (Figure 3A), a hallmark of mesenchymal cells. To validate whether the mutated-*EGFR* promotes vimentin expression, we further conducted immunohistochemistry (IHC) staining on clinical tumor samples. Correlation analysis revealed that the *EGFR*-mutation status is associated with vimentin expression as compared to WT-*EGFR* (75.3% *vs*. 51.2%, P = 0.007) (Figure 3G). This finding was further supported by the analysis of two different lung adenocarcinoma cohorts (Figure 3H) (The raw materials were download from publicly accessible datasets) [14–15]. These data indicate that the mutated-*EGFR* promotes lung cancer cell dissemination and correlates with vimentin expression.

### Mutated- *EGFR* NSCLC is associated with a shorter median time interval to SBM

To determine whether lung tumors harboring mutated-*EGFR*s are more aggressive than those with WT-*EGFR* in terms of SBM occurrence, we used a median time interval between the diagnosis of lung cancer and the detection of SBM (MTSBM) as a surrogate of tumor aggressiveness. As shown in Table 4, the mutated-*EGFR* tumor had a significantly shorter MTSBM than the WT-*EGFR* one did (for all-stage disease, 31.6 months *vs*. not reached (NR), P of log-rank test = 0.043; for stage IIIB-IV disease, 23.5 months *vs*. NR, P = 0.017). As to stage IIIB-IV diseases with mutated-*EGFR*s, the L858R mutation-positive tumors had a slightly shorter MTSBM compared to the exon 19 deletion-positive ones (22.9 *vs*. 26.4 months, P = 0.743), but the difference was not statistically different.

**Table 4:** The MTSBM in the patients without BM at the diagnosis of NSCLC

Patient group	Event of SBM / patient no.	MTSBM (month)	*P* of log-rank test
All stages			0.043
WT (ref.)	18 / 162	NR	
Mut	45 / 135	31.6	
Stage IIIB-IV			0.017
WT (ref.)	14 / 132	NR	
Mut	39 / 105	23.5	
Stage IIIB-IV, with mutated-EGFRs			0.743
Exon 19	17 / 43	26.4	
L858R (ref.)	20 / 58	22.9	

MTSBM: median time interval between the diagnosis of lung cancer and the detection of subsequent brain metastases; BM: brain metastases; NSCLC: non-small cell lung cancer; SBM: subsequent BM; Patient no.: patient number; WT: wild-type; Mut: mutated; *EGFR*: epidermal growth factor receptor; NR: not reached. The MTSBM was estimated using the Kaplan- Meier method and group difference (i.e., *EGFR* mutations) was compared using log-rank tests.

### *EGFR*-TKI administration is associated with SBM in mutated-*EGFR* patients

To determine whether *EGFR*-TKI treatment is associated with SBM, herein, we conducted a separate study. Of the patients without BM at the diagnosis of stage IIIB-IV NSCLC enrolled in Figure 2C right, 105 patients had mutated-*EGFRs*, 33 treated with *EGFR-*TKIs (TKI group) and 72 treated with non*-*TKI regimen (non-TKI group) as first-line treatment, respectively. More SBM was observed in TKI group (54.5%, 18/33) than in non-TKI group (29.2%, 21/72). As shown in Table 5, the administration of *EGFR*-TKIs as the 1^st^-line setting was associated with SBM (HR = 2.10, 95% CI = 1.15-3.82, P = 0.015) after adjusting for gender (HR = 2.38, 95% CI = 1.28-4.55, P = 0.007), age, smoking history and histological subtype.

**Table 5:** The association of the administration of *EGFR* -TKI as 1^st^t-line setting and subsequent BM occurrence among 105 stage IIIB-IV patients without BM at the diagnosis of mutated-*EGFR* NSCLC

Characteristics	Univariate		Multivariable	
	HR (95% CI)	*P*	HR (95% CI)	*P*
Gender (male / female)	0.65 (0.34–1.26)	0.201	0.85 (0.37–1.96)	0.704
Age (<60 /≧60)	2.44 (1.32–4.55)	0.005	2.38 (1.28–4.55)	0.007
Smoking (ever / never)	0.64 (0.29–1.40)	0.263	0.78 (0.29–2.15)	0.634
Histology (adenocarcinoma/non-adenocarcinoma)	0.56 (0.10–3.15)	0.509	0.66 (0.10–4.24)	0.660
1^st^-line TKI (yes / no)	2.11 (1.15–3.89)	0.016	2.10 (1.15–3.82)	0.015

*EGFR*: epidermal growth factor receptor; TKI: tyrosine kinase inhibitor; BM: brain metastasis; NSCLC: non-small cell lung cancer; HR: hazard ratio; CI: confidence interval. # The association between the administration of *EGFR*-TKI and subsequent BM was tested using a time-to-event analysis considering death as a competing risk (Fine and Gray's sub-distribution hazard model).

## DISCUSSION

The present study showed that the presence of mutated-*EGFRs* not only was associated with overall BM but also predicted SBM in stage IIIB-IV NSCLC, irrespective of the length of patient OS. NSCLC harboring mutated-*EGFRs* displayed a higher vimentin expression and had a significantly shorter MTSBM as compared to those with WT-*EGFR*. As to the subtype of mutated-*EGFR*s, the exon 19 deletion-positive and the L858R point mutation-positive patient groups shared a similar cumulative incidence of SBM.

The association between mutated-*EGFR*s and BM from NSCLC has been reported before but their conclusions were not consistent [3–6, 16–18], which could result from the differences in patient number and selection, statistical methodology or interventional treatments. Notably, the mutated-*EGFR* lung tumors were reported to be more sensitive to both cytotoxic chemotherapy and *EGFR-*TKIs than the wild-type ones [19]. Intriguingly, a better response to the treatment usually leads to a better disease control or a longer disease-free time interval at distant organs as well as a favorable survival; however, longer survival probably increases the risk of SBM development. Our analyses showed that a favorable OS was an important factor associated with SBM (P < 0.001, Figure 2A), which has been generally accepted and concerned, but was not clearly demonstrated in the previous reports [3–6, 16–18, 20, 21]. Notably, the rise of cumulative incidence curve of SBM was not apparent 3 years after the diagnosis of lung cancer (Figure 2C). Based on these results, the managements of SBM in NSCLC, including the prevention, early detection, and treatment, will become one of the main challenges for the patients who are expected to have a favorable survival, such as the mutated-*EGFR* group, especially during the first three-year follow-up.

We found that, independent of the survival factor, the presence of mutated-*EGFR*s was significantly associated with an increased risk of SBM in NSCLC as compared to those with WT-*EGFR* (HR = 2.98, P = 0.002). Similar results were recently reported by other research groups [4, 20, 21]. More importantly, we further observed that the lung tumors with mutated-*EGFR* progressed to the brain more rapidly than those with WT-*EGFR* in terms of MTSBM (23.5 months *vs*. NR, P = 0.017). These findings imply that the biological traits in cancer cells may contribute to SBM occurrence in NSCLC, however this possibility was rarely investigated in previous clinical reports. Prior *in vitro* experiments showed that the activation of *EGFR* upon ligand stimulation or by the mutation of *EGFR*vIII rather than *EGFR* overexpression correlates with cell migration and invasion in epithelial cancer cell lines, such as breast, oral squamous and glioblastoma cancers, and in NIH3T3 fibroblasts [11–13, 22]. Herein, we showed that the presence of mutated-*EGFR*s in lung cancer cells enhances cell mobility and promotes vimentin protein, a hallmark of mesenchymal cells [23]. Obviously, *in vitro* data in the current study were not robust and had limitations to indicate the presence of epithelial-to-mesenchymal transition (EMT). However, the additional analyses of tumor samples from our and others’ cohorts supported the correlation of *EGFR-*mutation status with vimentin expression, suggesting that *EGFR*-mutation status may be prone to undergo EMT-mediated cancer cell dissemination.

The varied treatments, including *EGFR-*TKIs, may affect the occurrence of SBM in mutated-*EGFR* patients. Some pilot studies demonstrated that first generation of *EGFR*-TKIs therapy impacted the development of BM progression in advanced NSCLC [24–26]. However, this issue was not further investigated nor concluded in the recently published reports [4–6, 20, 21], possibly due to limitation of the primary goals and designs of their studies. Our result showed that first generation of *EGFR-TKIs* treatment as 1^st^ setting is associated with SBM occurrence in mutated-*EGFR* patients (P = 0.015). One of the popular explanations for this phenomenon is that the discordance of drug concentration of *EGFR*-TKIs between in cerebrospinal fluid and in serum [27, 28], and the overall survival benefit of *EGFR*-TKIs provide chance and time for cancer cell colonization and proliferation in the brain, respectively. We acknowledged that the study population in Table 5 was small and highly selected, therefore large-scale studies are warranted to draw a firm conclusion in this issue. In addition, the authors also recognized that this finding could not be directly employed to the patients who receive third generation of *EGFR* TKIs (eg., Osimertinib, AZD3759), which has been reported to effectively penetrate the blood-brain barrier and display anti-tumor activity in the brain [29, 30].

Patients with an exon 19 deletion have longer survival compared to those with an *L858R* mutation [8–10], theoretically implying that more BM would be observed throughout the disease course of the former. One report supported this expectation (38.2% *vs*. 25.6%, P = 0.016) [4], but another [21] and our data did not, regarding the overall BM in the patients of all-stage disease. Our analysis further showed that the cumulative incidence of SBM in stage IIIB-IV NSCLC was similar between the *EGFR* exon 19 deletion-positive and the *L858R* mutation-positive groups (39.5%, *vs*. 34.5%), although the former exhibited a slightly longer OS (20.6 *vs*. 14.2 months). This finding may be partially explained by the observations that the L858R mutation-positive tumors had an inferior disease control rate to *EGFR-*TKIs as first-line treatment (63.6% *vs*. 100%, P of chi-square test = 0.017, table not shown).

**Figure 4:**
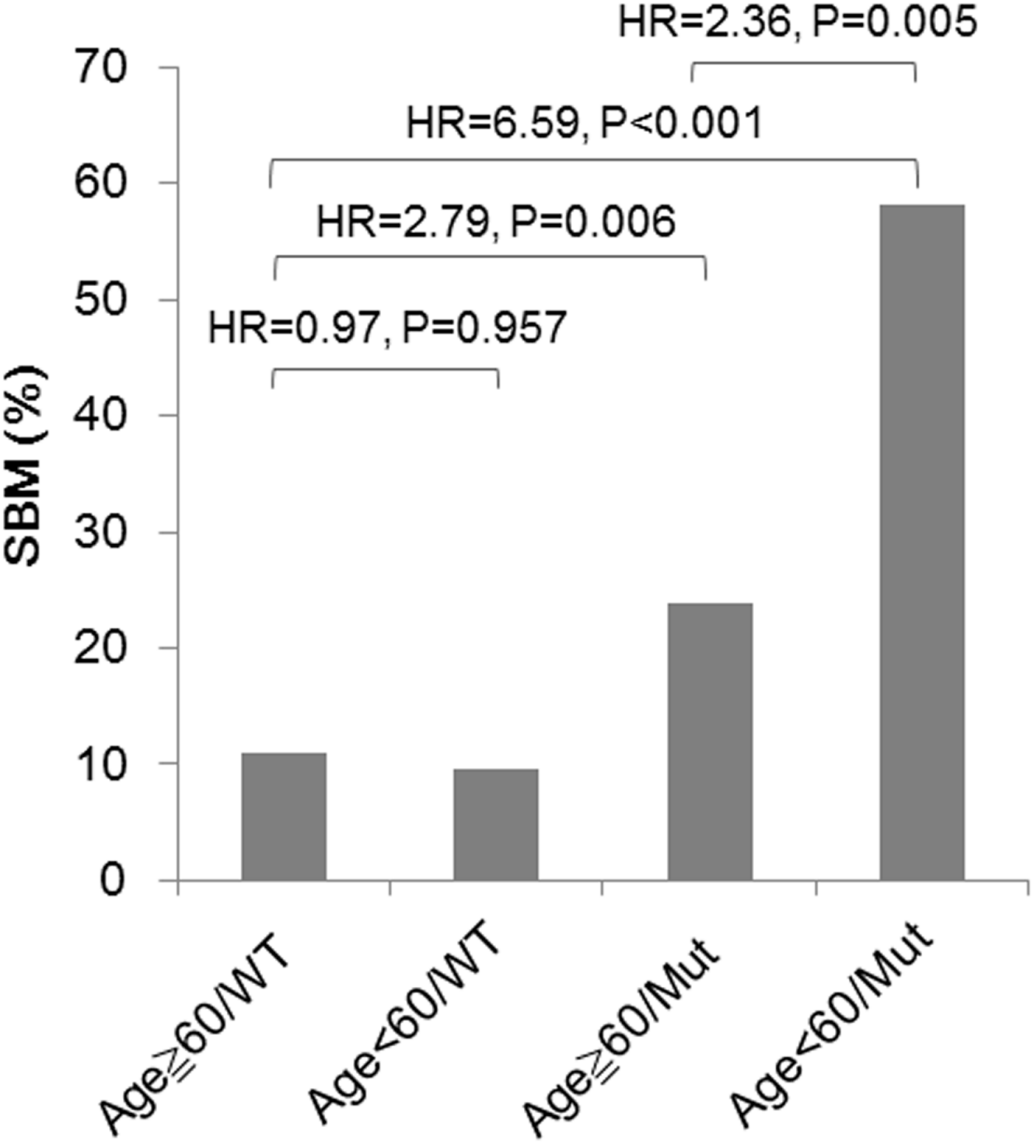
Risks for SBM in stage IIIB-IV NSCLC patients without BM at the time of lung cancer diagnosis, characterized by age and EGFR mutation status Comparison was conducted using a time-to-event analysis considering death as a competing risk (Fine and Gray's sub-distribution hazard model). P value < 0.05 was considered as significant.

One retrospective study suggested that a higher proportion of advanced mutated-*EGFR* NSCLC patients died of CNS metastases than did WT-*EGFR* patients (44.8% *vs*. 8.3%, P < 0.001) [31]. Our findings that several factors contributed to SBM in NSCLC may be helpful in better understanding SBM occurrence and in clinical practice. Stage IIIB-IV NSCLC patients without BM at the time of lung cancer diagnosis were classified into subgroups by age and *EGFR* mutation status, and their cumulative incidences of SBM varied widely from 10.9 % (11/101) to 58.1% (18/31) (Figure 4), which indicates that NSCLC patients with mutated-*EGFRs* may require a higher frequency of brain imaging assessments than those with WT-*EGFR* to facilitate earlier BM detection, especially in the subgroup characterized by younger age and mutated-*EGFR*s (HR = 6.57, 95% CI = 3.17-13.70, P < 0.001).

There are several limitations in the current study. First is the nature of the retrospective study; patient selection, especially the lump of histological subtypes of NSCLC, is a potential bias. Our additional analyses targeting the lung adenocarcinoma patients showed the comparable results (Supplementary Tables 1 and 2). Moreover, a higher proportion of patients with adenocarcinoma histology received the *EGFR* testing compared to those with SCC (71.4% *vs*. 39.6%) is the second limitation; however this limitation implies that the influence of the presence of mutated-*EGFR*s on SBM occurrence compared to that of WT-*EGFR* had been possibly underestimated because patients with lung SCC have a smaller chance of harboring mutated-*EGFR*s (1-3%) and of experiencing BM than those with lung adenocarcinoma [32]. The possibility of *EGFR* mutation discordance between the primary and metastatic sites may influence our results and represents another limitation. Based on the report indicating that the heterogeneous distribution of *EGFR* mutations is extremely rare in lung adenocarcinoma [33], we used the *EGFR* mutation status determined from primary or metastatic lung cancer specimens as a surrogate of the entire eligible population. Fourth, not all of the enrolled patients, such as those with early-stage disease, received intensively periodic brain imaging assessments after their NSCLC diagnosis. Furthermore, other driving oncogenes, such as *KRAS* and *ALK* mutations, were not factored into the analysis, largely because *KRAS* mutations were not common in our patient population (3.8%) [34] and *ALK* rearrangements were not routinely tested during the study period. Moreover, because few cases had the T790M mutation (n = 5) in our study, we could not address the potential influence of T790M on the BM development. In addition, we recognized that our findings were limited to externally generalize to NSCLC patients who visited and received their major managements for lung cancer at our institutes, not to the Taiwan population. All the previously mentioned limitations may have influenced the clinical findings in the current study. To further elucidate this issue, large-scale studies cross populations are warranted.

In summary, we showed that several factors contributed to SBM occurrence in NSCLC. A favorable OS correlated to a higher frequency of SBM. In addition, the presence of mutated-*EGFR*s predicted an increased risk for SBM independent of age and other common covariates, and was associated with a shorter MTSBM in stage IIIB-IV NSCLC patients. Furthermore, the presence of an *EGFR* exon19 deletion and the presence of an L858R point mutation are comparable to predict subsequent BM. These results suggest NSCLC patients with mutated-*EGFRs* may require a higher frequency of brain imaging assessments than those with WT-*EGFR* to facilitate earlier SBM detection during follow-up.

## MATERIALS AND METHODS

### Identification of the *EGFR* mutation status and BM in patients with NSCLC

Patients were selected from the lung cancer databases of Taipei Medical University Hospital (TMUH) and Wan Fang Hospital (WFH). Both TMUH, a teaching hospital, and WFH, a medical center, are run by Taipei Medical University. Patients were excluded if they had more than one primary cancer. Patients with stage I-IV NSCLC (including squamous cell carcinoma, adenocarcinoma, NSCLC-not otherwise specified and large cell carcinoma) that was histologically or cytologically confirmed between January 2006 and January 2012 (a total of 596, 382 cases from TMUH, 214 ones from WFH), and who had confirmed results from *EGFR* mutation testing were eligible for data collection by retrospective chart review (a total of 384, 195 cases from TMUH, 189 ones from WFH), with a data cutoff of August 2014 for outcome-SBM. This study was conducted with the approval of the Joint Institutional Review Board of Taipei Medical University (Approved number 201108006). Informed patient consent was obtained. The presence of BM was defined as previously described [35] (Supplementary Materials and Methods). *EGFR* exon 18-21 mutations were determined using direct DNA sequencing, as previously described [36] (Supplementary Materials and Methods). Patients with any or a combination of detectable *EGFR* exon 18-21 mutations, including common (exon 19 deletion and L858R point mutation), uncommon mutations (eg., exon 20 insertion, exon 18 mutation) and compound mutation (eg., exon 18/20), were placed into the mutated-*EGFR* group, and patients with no detectable mutated-*EGFRs* were put into the WT-*EGFR* group.

### *In vitro* assays

Human cell lines, including NCI-H1437 (ATCC CRL-5872), BEAS-2B (BCRC 60344) and HUVEC (BCRC H-UV001), were cultured for *in vitro* assays. The H1437 cells were kindly provided by Dr. Yu-Shan Chou at the Institute of Biomedical Sciences, Academia Sinica, Taiwan and further certified by the BCRC (Bioresource Collection and Research Center, Taiwan) through STR-PCR DNA profiling in 2014. The BEAS-2B cells were purchased from BCRC and certified via STR-PCR DNA profiling in 2017. The details of cell cultures, the plasmid construction, Q-PCR, and western blot analysis [37], wound healing and time-lapse cell tracking assays for the determination of cell motility conducted under the manufactures’ instruction, electric cell-substrate impedance sensing (ECIS) assay for the determination of cell barrier function and invasion ability [38, 39], and IHC were presented in the supplementary material method section (Supplementary Materials and Methods).

### Statistical analysis

The characteristics of the BM and non-BM patients were compared using Chi-square tests. The association between *EGFR* mutations (mutated *vs*. WT) and overall BM (BM at the diagnosis of lung cancer and SBM) was determined using a multivariable logistic regression analysis. For those NSCLC patients without BM at the diagnosis of lung cancer, we tested the association between the presence of mutated-*EGFR* and SBM using a time-to-event analysis considering death as a competing risk (Fine and Gray's sub-distribution hazard model). To compare the exon 19, L858R and WT groups, we conducted a separate model (both logistic and time-to-event models) incorporating the details of *EGFR*s. Regarding the covariate selection, only the covariates that were significant (*P* < 0.05) in the univariate model were included in the multivariable model. In addition to analyzing the NSCLC patients of all-stage disease, we also performed a subgroup analysis that only included patients of stage IIIB-IV disease. Therefore, we performed multiple tests involving four comparisons for each outcome (overall BM in Table 2 and SBM in Table 3). To prevent the problem of type-I error inflation, we set the alpha level to 0.05/4 = 0.0125 in the multivariable model (Tables 2 and 3). In addition, we depicted OS using the Kaplan-Meier method and compared group differences (i.e., *EGFR* mutations) using log-rank tests. Finally, the distribution of vimentin expression in lung cancer specimens was categorized into a dichotomous variable (positive *vs*. negative), and the difference between the mutated- and WT-*EGFR* groups was compared using Fisher's exact test. Generally, *P <* 0.05 was considered as significant. Data analyses were conducted using SPSS 22 (Armonk, NY: IBM Corp) and R 3.1.3 (R Core Team, Vienna, Austria).

## SUPPLEMENTARY MATERIALS FIGURES AND TABLES



## Abbreviations

BM: brain metastases
*EGFR*: epidermal growth factor receptor
CI: confidence interval
ECIS: electric cell-substrate impedance sensing
HR: hazard ratio
HUVEC: human umbilical vascular endothelial cells
MTSBM: median time interval between the diagnosis of lung cancer and the detection of SBM
NR: not reached
NSCLC: non-small cell lung cancer
OR: odds ratio
OS: overall survival
SBM: subsequent BM
TKD: tyrosine kinase domain
TKI: tyrosine kinase inhibitors
WT: wild-type

## References

[1] Mujoomdar A, Austin JH, Malhotra R, Powell CA, Pearson GD, Shiau MC, Raftopoulos H (2007). Clinical predictors of metastatic disease to the brain from non-small cell lung carcinoma: primary tumor size, cell type, and lymph node metastases. Radiology.

[2] Li T, Kung HJ, Mack PC, Gandara DR (2013). Genotyping and genomic profiling of non-small-cell lung cancer: implications for current and future therapies. J Clin Oncol.

[3] Doebele RC, Lu X, Sumey C, Maxson DA, Weickhardt AJ, Oton AB, Bunn PA, Barón AE, Franklin WA, Aisner DL, Varella-Garcia M, Camidge DR (2012). Oncogene status predicts patterns of metastatic spread in treatment-naive nonsmall cell lung cancer. Cancer.

[4] Shin DY, Na II, Kim CH, Park S, Baek H, Yang SH (2014). EGFR mutation and brain metastasis in pulmonary adenocarcinomas. J Thorac Oncol.

[5] Li B, Sun SZ, Yang M, Shi JL, Xu W, Wang XF, Song MM, Chen HM (2015). The correlation between EGFR mutation status and the risk of brain metastasis in patients with lung adenocarcinoma. J Neurooncol.

[6] Iuchi T, Shingyoji M, Itakura M, Yokoi S, Moriya Y, Tamura H, Yoshida Y, Ashinuma H, Kawasaki K, Hasegawa Y, Sakaida T, Iizasa T (2015). Frequency of brain metastases in non-small-cell lung cancer, and their association with epidermal growth factor receptor mutations. Int J Clin Oncol.

[7] Riely GJ, Pao W, Pham D, Li AR, Rizvi N, Venkatraman ES, Zakowski MF, Kris MG, Ladanyi M, Miller VA (2006). Clinical course of patients with non-small cell lung cancer and epidermal growth factor receptor exon 19 and exon 21 mutations treated with gefitinib or erlotinib. Clin Cancer Res.

[8] Yang JC, Wu YL, Schuler M, Sebastian M, Popat S, Yamamoto N, Zhou C, Hu CP, O'Byrne K, Feng J, Lu S, Huang Y, Geater SL (2015). Afatinib versus cisplatin-based chemotherapy for EGFR mutation-positive lung adenocarcinoma (LUX-Lung 3 and LUX-Lung 6): analysis of overall survival data from two randomised, phase 3 trials. Lancet Oncol.

[9] Lee CK, Wu YL, Ding PN, Lord SJ, Inoue A, Zhou C, Mitsudomi T, Rosell R, Pavlakis N, Links M, Gebski V, Gralla RJ, Yang JC (2015). Impact of specific epidermal growth factor receptor (EGFR) mutations and clinical characteristics on outcomes after treatment with EGFR tyrosine kinase inhibitors versus chemotherapy in EGFR-mutant lung cancer: a meta-analysis. J Clin Oncol.

[10] Karachaliou N, Mayo-de las Casas C, Queralt C, de Aguirre I, Melloni B, Cardenal F, Garcia-Gomez R, Massuti B, Sánchez JM, Porta R, Ponce-Aix S, Moran T, Carcereny E (2015). Association of EGFR L858R mutation in circulating free DNA with survival in the EURTAC trial. JAMA Oncol.

[11] Verbeek BS, Adriaansen-Slot SS, Vroom TM, Beckers T, Rijksen G (1998). Overexpression of EGFR and c-erbB2 causes enhanced cell migration in human breast cancer cells and NIH3T3 fibroblasts. FEBS Lett.

[12] Thomas SM, Coppelli FM, Wells A, Gooding WE, Song J, Kassis J, Drenning SD, Grandis JR (2003). Epidermal growth factor receptor-stimulated activation of phospholipase Cgamma-1 promotes invasion of head and neck squamous cell carcinoma. Cancer Res.

[13] Lo HW, Hsu SC, Xia W, Cao X, Shih JY, Wei Y, Abbruzzese JL, Hortobagyi GN, Hung MC (2007). Epidermal growth factor receptor cooperates with signal transducer and activator of transcription 3 to induce epithelial-mesenchymal transition in cancer cells via up-regulation of TWIST gene expression. Cancer Res.

[14] Chitale D, Gong Y, Taylor BS, Broderick S, Brennan C, Somwar R, Golas B, Wang L, Motoi N, Szoke J, Reinersman JM, Major J, Sander C (2009). An integrated genomic analysis of lung cancer reveals loss of DUSP4 in EGFR-mutant tumors. Oncogene.

[15] Cancer Genome Atlas Network Comprehensive molecular profiling of lung adenocarcinoma. Nature.

[16] Hendriks LE, Smit EF, Vosse BA, Mellema WW, Heideman DA, Bootsma GP, Westenend M, Pitz C, de Vries GJ, Houben R, Grünberg K, Bendek M, Speel EJ (2014). EGFR mutated non-small cell lung cancer patients: more prone to development of bone and brain metastases?. Lung Cancer.

[17] Rangachari D, Yamaguchi N, VanderLaan PA, Folch E, Mahadevan A, Floyd SR, Uhlmann EJ, Wong ET, Dahlberg SE, Huberman MS, Costa DB (2015). Brain metastases in patients with EGFR-mutated or ALK-rearranged non-small-cell lung cancers. Lung Cancer.

[18] Baek MY, Ahn HK, Park KR, Park HS, Kang SM, Park I, Kim YS, Hong J, Sym SJ, Park J, Lee JH, Shin DB, Cho EK (2016). Epidermal growth factor receptor mutation and pattern of brain metastasis in patients with non-small cell lung cancer. Korean J Intern Med.

[19] Mok TS, Wu YL, Thongprasert S, Yang CH, Chu DT, Saijo N, Sunpaweravong P, Han B, Margono B, Ichinose Y, Nishiwaki Y, Ohe Y, Yang JJ (2009). Gefitinib or carboplatin-paclitaxel in pulmonary adenocarcinoma. N Engl J Med.

[20] Hsu F, De Caluwe A, Anderson D, Nichol A, Toriumi T, Ho C (2016). EGFR mutation status on brain metastases from non-small cell lung cancer. Lung Cancer.

[21] Han G, Bi J, Tan W, Wei X, Wang X, Ying X, Guo X, Zhou X, Hu D, Zhen W (2016). A retrospective analysis in patients with EGFR-mutant lung adenocarcinoma: is EGFR mutation associated with a higher incidence of brain metastasis?. Oncotarget.

[22] Lal A, Glazer CA, Martinson HM, Friedman HS, Archer GE, Sampson JH, Riggins GJ (2000). Mutant epidermal growth factor receptor up-regulates molecular effectors of tumor invasion. Cancer Res.

[23] De Craene B, Berx G (2013). Regulatory networks defining EMT during cancer initiation and progression. Nat Rev Cancer.

[24] Omuro AM, Kris MG, Miller VA, Franceschi E, Shah N, Milton DT, Abrey LE (2005). High incidence of disease recurrence in the brain and leptomeninges in patients with nonsmall cell lung carcinoma after response to gefitinib. Cancer.

[25] Lee YJ, Choi HJ, Kim SK, Chang J, Moon JW, Park IK, Kim JH, Cho BC (2010). Frequent central nervous system failure after clinical benefit with epidermal growth factor receptor tyrosine kinase inhibitors in Korean patients with nonsmall-cell lung cancer. Cancer.

[26] Heon S, Yeap BY, Britt GJ, Costa DB, Rabin MS, Jackman DM, Johnson BE (2010). Development of central nervous system metastases in patients with advanced non-small cell lung cancer and somatic EGFR mutations treated with gefitinib or erlotinib. Clin Cancer Res.

[27] Pitz MW, Desai A, Grossman SA, Blakeley JO (2011). Tissue concentration of systemically administered antineoplastic agents in human brain tumors. J Neurooncol.

[28] Togashi Y, Masago K, Masuda S, Mizuno T, Fukudo M, Ikemi Y, Sakamori Y, Nagai H, Kim YH, Katsura T, Mishima M (2012). Cerebrospinal fluid concentration of gefitinib and erlotinib in patients with non-small cell lung cancer. Cancer Chemother Pharmacol.

[29] Ballard P, Yates JW, Yang Z, Kim DW, Yang JC, Cantarini M, Pickup K, Jordan A, Hickey M, Grist M, Box M, Johnström P, Varnäs K (2016). Preclinical comparison of osimertinib with other EGFR-TKIs in EGFR-mutant NSCLC brain metastases models, and early evidence of clinical brain metastases activity. Clin Cancer Res.

[30] Zeng Q, Wang J, Cheng Z, Chen K, Johnström P, Varnäs K, Li DY, Yang ZF, Zhang X (2015). Discovery and evaluation of clinical candidate AZD3759, a potent, oral active, central nervous system-penetrant, epidermal growth factor receptor tyrosine kinase inhibitor. J Med Chem.

[31] Wu WS, Chen YM, Tsai CM, Shih JF, Lee YC, Perng RP, Whang-Peng J (2013). The epidermal growth factor receptor-tyrosine kinase inhibitor era has changed the causes of death of patients with advanced non-small-cell lung cancer. J Chin Med Assoc.

[32] Mamon HJ, Yeap BY, Jänne PA, Reblando J, Shrager S, Jaklitsch MT, Mentzer S, Lukanich JM, Sugarbaker DJ, Baldini EH, Berman S, Skarin A, Bueno R (2005). High risk of brain metastases in surgically staged IIIA non-small-cell lung cancer patients treated with surgery, chemotherapy, and radiation. J Clin Oncol.

[33] Yatabe Y, Matsuo K, Mitsudomi T (2011). Heterogeneous distribution of EGFR mutations is extremely rare in lung adenocarcinoma. J Clin Oncol.

[34] Wu CC, Hsu HY, Liu HP, Chang JW, Chen YT, Hsieh WY, Hsieh JJ, Hsieh MS, Chen YR, Huang SF (2008). Reversed mutation rates of KRAS and EGFR genes in adenocarcinoma of the lung in Taiwan and their implications. Cancer.

[35] Hsiao SH, Chung CL, Chou YT, Lee HL, Lin SE, Liu HE (2013). Identification of subgroup patients with stage IIIB/IV non-small cell lung cancer at higher risk for brain metastases. Lung Cancer.

[36] Hsiao SH, Chung CL, Lee CM, Chen WY, Chou YT, Wu ZH, Chen YC, Lin SE (2013). Suitability of computed tomography-guided biopsy specimens for subtyping and genotyping of non-small-cell lung cancer. Clin Lung Cancer.

[37] Chou YT, Lin HH, Lien YC, Wang YH, Hong CF, Kao YR, Lin SC, Chang YC, Lin SY, Chen SJ, Chen HC, Yeh SD, Wu CW (2010). EGFR promotes lung tumorigenesis by activating miR-7 through a Ras/ERK/Myc pathway that targets the Ets2 transcriptional repressor ERF. Cancer Res.

[38] Tiruppathi C, Malik AB, Del Vecchio PJ, Keese CR, Giaever I (1992). Electrical method for detection of endothelial cell shape change in real time: assessment of endothelial barrier function. Proc Natl Acad Sci U S A.

[39] Keese CR, Bhawe K, Wegener J, Giaever I (2002). Real-time impedance assay to follow the invasive activities of metastatic cells in culture. Biotechniques.

