# Supplementary material for: Brain metastases in patients with non-small cell lung cancer: the role of mutated-EGFRs with an exon 19 deletion or L858R point mutation in cancer cell dissemination
Source: Oncotarget. 2017 Jun 16;8(32):53405–18. doi: 10.18632/oncotarget.18509 (PMC5581119; doi:10.18632/oncotarget.18509)
Supplement: Supplementary file 1 [file oncotarget-08-53405-s001.pdf]

Brain metastases in patients with non-small cell lung cancer: the role of mutated-*EGFRs* with an exon 19 deletion or L858R point mutation in cancer cell dissemination

SUPPLEMENTARY MATERIALS AND METHODS

Detection of brain metastases (BM)

All of the enrolled all-stage patients of were imaged to detect BM at the time of their lung cancer diagnosis (baseline). BM was defined as the presence of one or more intra-axial enhancing lesions on gadolinium-enhanced brain magnetic resonance imaging (MRI) or contrast-enhanced computed tomography (CT) images of the head. Leptomeningeal metastases were not included in the actuarial incidence of BM in this study. MRI is preferred for the evaluation of BM in our setting, and CT was used to replace MRI in some patients who were contraindicated to MRI or who could not tolerate the process of MRI. Typically, for stage IIIB-IV patients, they received a 3-month interval follow-up study to assess systemic control of their disease, including a CT scan of the chest, and a brain MRI, which was largely based on the reimbursement policy in our country. Stage I-IIIA patients were imaged to detect subsequent BM when BM-associated neurologic symptoms/signs emerged, when the level of tumor markers, such as CEA, increased beyond the normal limit, or when they received their annual post-operation follow-up. Generally, the post-operative follow-up continued for five years after surgery.

EGFR mutation test

An *EGFR* mutation test was conducted at the request of clinicians from January 2006 to May 2011, and after June 2011, the test was routinely performed at the time of lung adenocarcinoma diagnosis in accordance with the reimbursement policy in our country. The majority of the tumor specimens used for the *EGFR* tests were small-biopsied samples in the current study and subsequent tumor specimens were extremely rare because re-biopsy after treatment failure was not widely recommended during the study period (Jan. 2006 to Jan. 2012). Histological specimens submitted for *EGFR* mutation testing were reviewed and evaluated for the suitability of further molecular analyses by 1 pulmonary pathologist (either SEL or WYC). Specifically, cancer cells representing less than 25% of overall tumor tissue were determined as not suitable for molecular testing. Typically, genomic DNA was extracted from 3 suitable 5-μm thick section cuts from the formalin-fixed, paraffin-embedded tumor tissue, and *EGFR* exons 18 to 21 were amplified using polymerase chain reaction and analyzed bidirectionally using direct DNA sequencing for the presence of somatic mutations according to previously described methods. Then, analyses of the DNA sequences were performed in both forward and reverse directions (Hsiao SH, et al. Clinical Lung Cancer 2013).

Treatment history

During the period from Jan. 2006 to May 2011, the majority of the enrolled stage IIIB-IV patients received cytotoxic chemotherapy as the first-line standard of care for their illness. After June 2011, targeted therapy (*EGFR*-tyrosine kinase inhibitors, *EGFR*-TKIs) has been the standard of the first-line care for stage IIIB-IV lung adenocarcinoma with mutated-*EGFRs* in our country. Specifically, 105 of 237 stage IIIB-IV non-small cell lung cancer (NSCLC) patients, who did not have BM at the diagnosis of lung cancer, had mutated-*EGFRs*; 33 (31.4%) of them received *EGFR*-TKIs (the first-generation), and the remaining received chemotherapy as first-line treatment for their illness. Of the stage I-IIIA NSCLC patients enrolled in the current study, 40 received a curative surgery as the primary treatment after a thorough preoperative evaluation.

In vitro assays

Cell culture. Human cell lines, including NCI-H1437 (ATCC CRL-5872), BEAS-2B (BCRC 60344) and HUVEC (BCRC H-UV001), were cultured for *in vitro* assays. The H1437 cells (human lung adenocarcinoma wild-type *EGFR* cell line) were kindly provided by Dr. Yu-Shan Chou at the Institute of Biomedical Sciences, Academia Sinica and certified by the BCRC (Bioresource Collection and Research Center, Taiwan) through STR-PCR DNA profiling in 2014, and cultured in RPMI-1640 (Sigma-Aldrich, MO, USA), supplemented with 10% fetal bovine serum (FBS) at 37°C in 5% CO_2. HUVECs were purchased from and certified by the BCRC, and grown in Medium 199 with 25 U/ml heparin, 30 μg/ml endothelial cell growth supplement and 10% FBS. The BEAS-2B cells (human bronchial epithelia cells) were purchased from the BCRC and cultured in Ham's F12 medium with 1.5 g/L

sodium bicarbonate, 2.7 g/L glucose, 2.0 mM L-glutamine, 0.1 mM nonessential amino acids, 0.005 mg/ml insulin, 10 ng/ml epidermal growth factor, 0.001 mg/ml transferrin, 500 ng/ml hydrocortisone and 4% FBS. The BEAS-2B cells were certified by the BCRC in 2017.

DNA plasmid generation

To generate the pHR′-puro-*EGFR* wild-type (WT), L858R and Del 3 plasmids, pBabe *EGFR* WT (ID: 11011), pBabe *EGFR* L858R (ID: 11012) and pBabe *EGFR* (Del 3) L747-E749del, A750P (ID: 11015) were purchased from Addgene (Cambridge, MA, USA) and the respective segments containing the *EGFR* WT, L858R and del coding regions (digested with BamHI and SalI) were inserted into the pHR′-puro lentiviral vector. The pHR′-puro (Ctrl) lentiviral expression vector has been described previously. Virus production and infection were performed as described.

Q-PCR

The total RNAs were extracted using Trizol (Invitrogen, CA, USA) according to the manufacturer's instructions. The first-strand cDNAs were synthesized with the PrimeScript RT reagent Kit (Takara, Shiga, Japan) using 1 μg of RNA according to the manufacturer's protocol. Quantitative-PCR was performed as described. The relative gene expression was analyzed with the 2-ΔΔCT method using the LightCycler 480 Real-Time PCR System (Roche Applied Science, Mannheim, Germany) combined with the Universal Probe Library (Roche Applied Science). The 18S rRNA was used as a reference transcript. The primer sequences used to detect specific genes are listed below.

Western blot analysis

Western blotting was performed as described. The primary antibodies, including *EGFR* (1902-1, Epitomics), p*EGFR* (3777, Cell Signaling Technology), Vimentin (A1504, Abclonal Biotech), and GAPDH (GTX100118, GeneTex), and HRP-conjugated secondary antibodies from Santa Cruz Biotechnology (1:5000) (Santa Cruz, CA, USA) were used.

Immunohistochemistry (IHC)

IHC was performed using the anti-Vimentin antibody (V9, Dako, CA, USA). The staining intensity was determined by cytosolic staining. Vimentin expression in equal to or more than 10% of cancer cells was categorized as positive. Vimentin-positive staining in the lung adenocarcinoma specimens was recorded with an Olympus photo system (Olympus DP20 microscope camera) and examined by one molecular pathologist (S.E.L.).

Wound healing and time-lapse cell tracking (TLCT) assay

Wound healing and TLCT assays were performed according to the manufacturer's (Leica DMI 6000B) instructions. In the TLCT assay, the cells were seeded in 6-well plates at 2000 cells/well and cell migration was tracked by time-lapse microscopy over 24 h. The results of the TLCT assay were analyzed using MetaMorph® software. The difference of cell migration / movements between cancer cells with mutated-*EGFR*s (Del3 and L858R) and WT-*EGFR* cancer cells was analyzed using student *t*-test.

Electric cell-substrate impedance sensing (ECIS) assay

1. An ECIS device (ECIS 8Z; Applied Biophysics, Troy, NY) was used in this study. The details of ECIS assays have been described previously (Ref. 17, 18). The principles behind ECIS assays are briefly described below. The impedance (Z) is determined by Ohm's law Z =V/I. When cells are added to the ECIS® Arrays and attached to the electrodes, they act as insulators, thus increasing the impedance. As cells grow and cover the electrodes, the current is impeded in a manner that is related to the number of cells covering the electrode, the morphology of the cells and the nature of the cell attachment (Products Guide, Applied Biophysics, 2012).
2. In the current study, the impedance was monitored to check confluence. The electrode arrays consisted of eight individual small electrodes with larger counter electrodes located at the base of small wells for cell culture (8W10E; Applied Biophysics, Inc.). H1437/WT/L858R/Del3 cell suspensions (5000 cells per well) were separately seeded into the wells, and the impedance measure was monitored to check confluence maintained at 4000 Hz for the next 6 hours.
3. To determine the invasion capacity, an endothelial cell monolayer covering the electrodes was established using a HUVEC suspension that was added to each well (20000 cells per well) 12 hours before the H1437/WT/L858R/Del3 cells were separately seeded into the wells (5000 cells per well). The resistive portion of impedance (TER), normalized to its initial value at time 0, was monitored continuously and used as a measure of barrier integrity. Change in TER, which is a measure of the endothelial barrier integrity

of the HUVEC monolayer, was determined using ECIS and reflected the changes in HUVEC barrier function in response to the cancer cells. To compare the experimental conditions, data were normalized to the mean resistance of each condition when the monolayer had a constant resistance immediately prior to stimuli addition.

Public domain dataset analyses

The sources of *VIM* expression levels of lung adenocarcinoma and the corresponding clinicopathological parameters were downloaded from Chitale et.al. : Memorial Sloan-Kettering Cancer Center (http://cbio.mskcc.org/public/lung_array_data/) and TCGA (https://tcga-data.nci.nih.gov/docs/publications/luad_2014/) (Chi-square test).

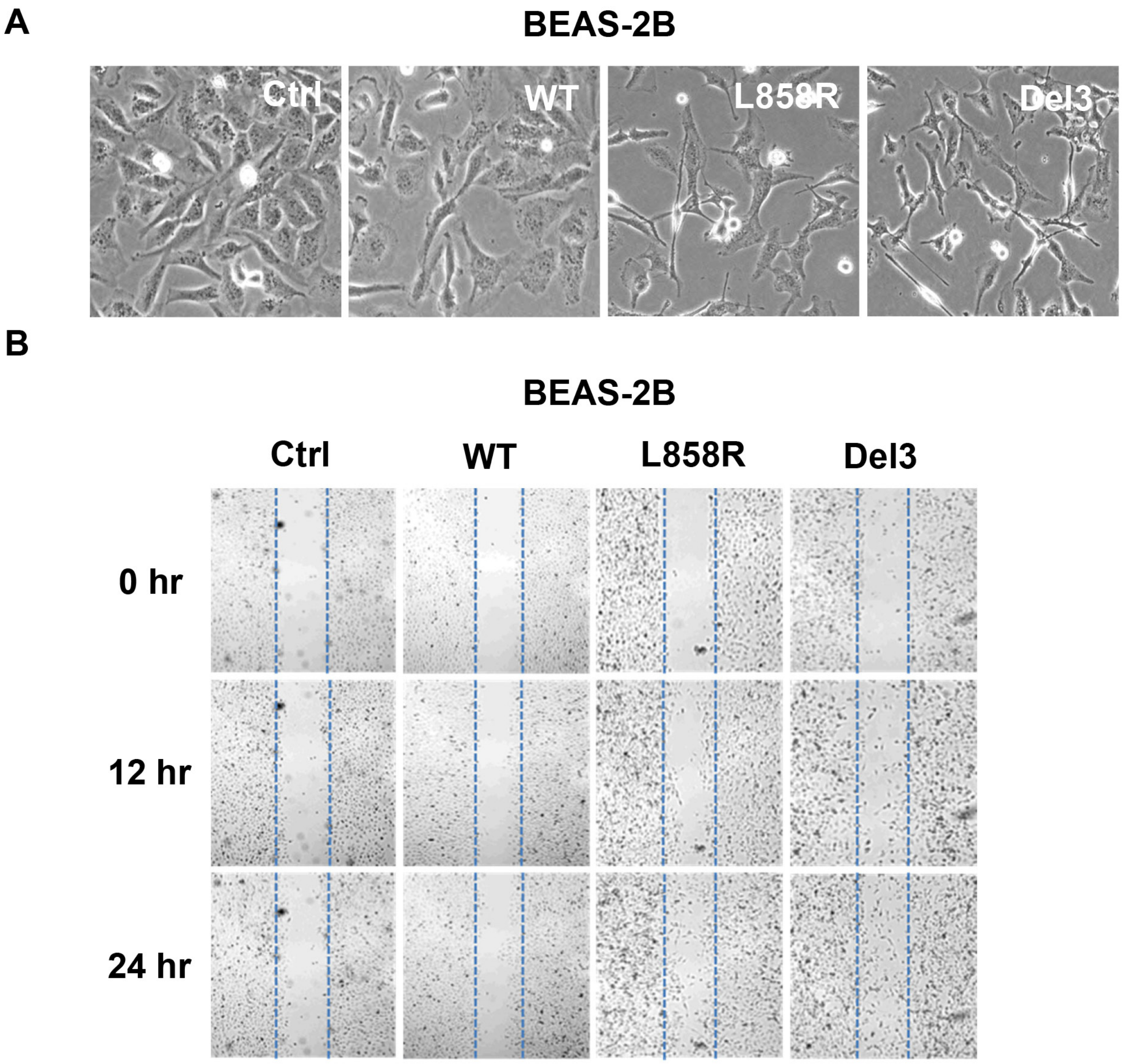

Supplementary Figure 1: *EGFR*-mutations promote cell migration. (A) Representative phase-contrast images of BEAS2B (non-tumorous lung epithelial cells) infected with lentiviral vectors encoding WT and mutated-*EGFR*s (L858R or Del 3) or empty control vector. **(B)** Wound-healing analysis to assess the cell mobility of BEAS2B infected with lentiviral vectors encoding WT and mutated-*EGFR*s (L858R or Del 3) or empty control vector.

Supplementary Table 1: Multivariable logistic regression analysis of clinical variables for overall BM among 320 patients with lung adenocarcinoma

Characteristics	All patients (*N* = 320)				Stage IIIB - IV (*n* = 269)			
	Univariate		Multivariable		Univariate		Multivariable	
	OR (95% CI)	P	OR (95% CI)	P	OR (95% CI)	P	OR (95% CI)	P
Gender (male / female)	0.61 (0.39–0.96)	0.031	0.76 (0.42–1.39)	0.373	0.45 (0.28–0.73)	0.001	0.59 (0.32–1.11)	0.103
Age (<60 /≧60)	2.63 (1.61–4.35)	<0.001	2.63 (1.54–4.35)	<0.001	2.63 (1.52–4.55)	0.001	2.44 (1.41–4.34)	0.002
Smoking (ever / never)	0.53 (0.33–0.85)	0.008	0.74 (0.39–1.40)	0.349	0.45 (0.27–0.74)	0.002	0.79 (0.41–1.54)	0.493
Stage (IIIB-IV/ I-IIIA)	3.11 (1.53–6.32)	0.002	3.65 (1.74–7.66)	0.001	–	–	–	–
EGFR (Mut / WT)	2.21 (1.39–3.49)	0.001	1.95 (1.18–3.24)	0.010	2.56 (1.56–4.21)	<0.001	2.04 (1.20–3.47)	0.009
Pairwise comparison								
Exon 19 / WT	2.22 (1.24–3.95)	0.007	1.74 (0.92–3.29)	0.090	2.77 (1.46–5.23)	0.002	1.95 (0.99–3.85)	0.055
L858R / WT	2.16 (1.26–3.68)	0.005	2.02 (1.14–3.61)	0.017	2.40 (1.35–4.27)	0.003	2.10 (1.14–3.86)	0.017
Exon 19 / L858R	1.03 (0.56–1.89)	0.930	0.86 (0.45–1.65)	0.648	1.15 (0.59–2.26)	0.682	0.93 (0.46–1.88)	0.836

EGFR: epidermal growth factor receptor; Mut: mutated; WT: wild-type; # Uncommon mutation was not shown in the *EGFR* pairwise comparison due the small number of patients. The association between *EGFR* mutations (mutated *vs*. WT) and BM (BM at time of lung cancer diagnosis and subsequent BM) was determined using a multivariable logistic regression analysis. In addition, the pairwise comparisons were made in another multivariable logistic regression model; OR: odds ratio; CI: confidence interval.

Supplementary Table 2: Multivariable analysis of the clinical factors for the occurrence of the subsequent BM among 244 patients without BM at the diagnosis of their lung adenocarcinoma

Characteristics	All adenocarcinoma patients (*N* = 244)				Stage IIIB - IV (*n* = 194)			
	Univariate		Multivariable		Univariate		Multivariable	
	HR (95% CI)	*P*	HR (95% CI)	*P*	HR (95% CI)	*P*	HR (95% CI)	*P*
Gender (male / female)	0.82 (0.49–1.36)	0.440			0.57 (0.32–0.99)	0.049	0.71 (0.40–1.23)	0.220
Age (<60 /≧60)	2.56 (1.56–4.35)	<0.001	2.50 (1.49–4.17)	<0.001	2.44 (1.41–4.35)	0.002	2.22 (1.28–3.85)	0.004
Smoking (ever / never)	0.65 (0.37–1.14)	0.140			0.54 (0.29–1.00)	0.052		
Stage (IIIB-IV / I-IIIA)	1.40 (0.74–2.65)	0.300			–	–	–	–
EGFR (Mut / WT)	2.32 (1.31–4.11)	0.004	2.23 (1.26–3.96)	0.006	2.95 (1.54–5.65)	0.001	2.57 (1.34–4.94)	0.005
Pairwise comparison								
Exon 19 / WT	2.13 (1.09–4.15)	0.027	1.80 (0.92–3.53)	0.087	2.89 (1.38–6.08)	0.005	2.14 (1.00–4.55)	0.049
L858R / WT	2.32 (1.22–4.42)	0.011	2.46 (1.29–4.68)	0.006	2.89 (1.41–5.93)	0.004	2.89 (1.41–5.94)	0.004
Exon 19 / L858R	0.92 (0.50–1.70)	0.790	0.73 (0.39–1.36)	0.320	1.00 (0.53–1.91)	0.990	0.74 (0.38–1.45)	0.380

BM: brain metastases; *EGFR*: epidermal growth factor receptor; Mut: mutated; WT: wild type; HR: hazard ratio; CI: confidence interval.
Uncommon mutation was not shown in the *EGFR* pairwise comparison due to the small number of patients. In addition–the pairwise comparisons were made in another multivariable model. The association between the presence of mutated-*EGFR* and subsequent BM was tested using a time-to-event analysis considering death as a competing risk (Fine and Gray's sub-distribution hazard model).
